# Effect of tissue permeability and drug diffusion anisotropy on convection-enhanced delivery 

**DOI:** 10.1080/10717544.2019.1639844

**Published:** 2019-07-30

**Authors:** Wenbo Zhan, Ferdinando Rodriguez y Baena, Daniele Dini

**Affiliations:** Department of Mechanical Engineering, Imperial College London, London, UK

**Keywords:** Anisotropy, convection-enhanced delivery, drug transport, mathematical model

## Abstract

Although convection-enhanced delivery (CED) can successfully facilitate a bypass of the blood brain barrier, its treatment efficacy remains highly limited in clinic. This can be partially attributed to the brain anisotropic characteristics that lead to the difficulties in controlling the drug spatial distribution. Here, the responses of six different drugs to the tissue anisotropy are examined through a parametric study performed using a multiphysics model, which considers interstitial fluid flow, tissue deformation and interlinked drug transport processes in CED. The delivery outcomes are evaluated in terms of the penetration depth and delivery volume for effective therapy. Simulation results demonstrate that the effective penetration depth in a given direction can be improved with the increase of the corresponding component of anisotropic characteristics. The anisotropic tissue permeability could only reshape the drug distribution in space but has limited contribution to the total effective delivery volume. On the other hand, drugs respond in different ways to the anisotropic diffusivity. The large delivery volumes of *fluorouracil*, *carmustine*, *cisplatin* and *doxorubicin* could be achieved in relatively isotropic tissue, while *paclitaxel* and *methotrexate* are able to cover enlarged regions into anisotropic tissues. Results obtained from this study serve as a guide for the design of CED treatments.

## 1. Introduction

Routine drug delivery into brain through intravenous administration is less effective in clinic, mainly owing to the blood–brain barrier that can successfully retard the drug transvascular transport (Zhou et al., 2013). Convection-enhanced delivery (CED) has been developed as an alternative to directly infuse the drug solution into the lesion. The enhanced bulk flow of interstitial fluid is expected to improve the drug penetration into deep tissue for better therapy. The feasibility and safety of CED has been extensively reported in the literature (Brady et al., 2011); however, its applications in clinical trials for treating Parkinson’s disease (Marks et al., 2010) and brain cancers (Groothuis, 2000) remain to be optimized and results so far have been somewhat disappointing. Follow-up analyses demonstrated that the insufficient drug accumulation and difficulties to control the drug spatial distribution are the main barriers to be overcome for a better uptake of CED in clinical treatments (Raghavan et al., 2006; Marks et al., 2010; Grondin et al., 2019).

Intracerebral drug delivery involves multiple biophysical and physicochemical processes determined by drug transport properties, biological properties of the brain and their interplays. Brain tissue is highly anisotropic due to the wide distribution of nerve fibers, resulting in the significant variations of tissue permeability and drug diffusivity in different directions which were found to strongly influence drug transport (Zuzana & Syková, 1998). Given the anisotropy could change in a large range depending on the location, organization and distribution of nerve fibers, examining the performances of various drugs under different anisotropic conditions could deepen understanding on the effects of anisotropic properties on CED treatments and optimize the treatment planning.

Numerical simulation has become a promising approach to study drug delivery because of its advantage in examining the complex drug transport processes integrally or in an individual manner (Sefidgar et al., 2014; Steuperaert et al., 2017; Zhan et al., 2019; Manshadi et al., 2019). The modeling framework in the general form was firstly setup to study the intravenous administration of antibodies in different *in vivo* environments (Baxter & Jain, 1989, 1990, 1991) and was further tailored to predict drug transport in CED treatments (Støverud et al., 2012; Zhang et al., 2012). Brain was simplified as an isotropic domain in most of the previous studies owing to the lack of supports from the experimental measurements (Arifin et al., 2009; Zhang et al., 2009). As an improvement, a calibration algorithm was developed to derive the brain anisotropy from dMRI data (Linninger et al., 2008) and has been applied in the study to understand the influence of particular infusion locations on the intracerebral transport of trophic factor.

In the present study, a multiphysics model is applied to a 2D axis-symmetric geometry with the aim to examine the impacts of anisotropic characterization on the delivery of different drugs upon CED. The model covers the key drug delivery processes; these include convective transport with interstitial fluid flow, diffusion in the tissue interstitial space, binding with proteins, cell uptake, and elimination due to blood drainage, metabolism and physical degradation. The delivery outcomes are evaluated in terms of the penetration depth and delivery volume in which the concentration of each drug is greater than its LD_90_.

## 2. Material and methods

### 2.1. Mathematical model

Given that the inter-capillary distance is orders lower than the drug transport dimension, brain tissue is commonly treated as a porous medium (Baxter & Jain, 1989), which is fully saturated with the incompressible, Newtonian interstitial fluid. The infinitesimal tissue deformation during CED infusion is described by the linear solid mechanics model (Su et al., 2011) in the form of

(1) $$G\nabla^{2}u+\left(\lambda +G\right)\nabla \left(\nabla \cdot u\right)=\nabla \left(\upsilon_{\mathrm{IS}}p_{i}\right)$$

where **u** stands for the tissue displacement vector, and *λ* and *G* are tissue Lamé constants. $\upsilon_{\mathrm{IS}}$ refers to the fraction of tissue interstitial space which is determined by the tissue deformation as $\upsilon_{\mathrm{IS}}=\left(\upsilon_{\mathrm{IS},0}+e\right)/\left(1+e\right),$ where e=∇·u, and $\upsilon_{\mathrm{IS},0}$ refers to the initial value. *p_i_* is the interstitial fluid pressure that is governed by the mass and momentum conservation equations (Baxter & Jain, 1989), as follows:

(2) $$\begin{array}{c} \nabla \cdot v=L_{b}\frac{S}{V}\left[p_{b}-p_{i}-\sigma_{T}\left(\pi_{b}-\pi_{i}\right)\right]\\ \rho \left(v\cdot \nabla v\right)=-\upsilon_{\mathrm{IS}}\nabla p_{i}+\mu \nabla^{2}v-\upsilon_{\mathrm{IS}}\frac{\mu }{\kappa }v \end{array}$$

where *ρ* and *μ* are the interstitial fluid density and viscosity, respectively, and **v** refers to the interstitial fluid velocity. *L_b_* is the hydraulic conductivity of blood vessel wall, and *S/V* is the area of blood vessel per tissue volume. *p_b_* is the blood pressure. *π_b_* and *π_i_* denote the osmotic pressure of blood and interstitial fluid, respectively. *κ* is the tissue permeability which is governed by $\kappa =\kappa_{0}e\mathrm{xp}\left(\mathrm{Me}\right)$ upon tissue deformation, where *M* is a non-dimensional parameter describing the properties of tissue extracellular matrix and *κ*_0_ is the initial tissue permeability before CED infusion takes place (Su et al., 2011).

The brain tissue can briefly be divided into three compartments, including interstitial space (IS), cell membrane (CM) and cell interior (CI). The concentration (*C*) of free drugs (F) and drugs that bind with proteins (B) are governed by the mass conservation equations as

(3) $$\begin{array}{c} C_{F}=\upsilon_{\mathrm{IS}}C_{F,\mathrm{IS}}+\upsilon_{\mathrm{CI}}C_{F,\mathrm{CI}}+\upsilon_{\mathrm{CM}}C_{F,\mathrm{CM}}\\ C_{B}=\upsilon_{\mathrm{IS}}C_{B,\mathrm{IS}}+\upsilon_{\mathrm{CI}}C_{B,\mathrm{CI}}+\upsilon_{\mathrm{CM}}C_{B,\mathrm{CM}} \end{array}$$

where *υ* refers to the volume fraction. It is assumed that there is no drug either being eliminated or associated with proteins on CM (Arifin et al., 2009). Free drug accumulation in the whole brain is determined by convective and diffusive transport in IS, binding with proteins, cell uptake and elimination due to the loss to blood circulatory system, physical degradation and metabolism. Therefore, the concentration of free drugs in the entire tissue is described by

(4) $$\frac{\partial C_{F}}{\partial t}=\upsilon_{\mathrm{IS}}{D_{\mathrm{IS}}\nabla }^{2}C_{F,\mathrm{IS}}-\nabla \cdot \left(\upsilon_{\mathrm{IS}}vC_{F,\mathrm{IS}}\right)-\upsilon_{\mathrm{IS}}\left(k_{b}+k_{e}\right)C_{F,\mathrm{IS}}-\upsilon_{\mathrm{CI}}k_{e}C_{F,\mathrm{CI}}-\frac{\partial C_{B}}{\partial t}$$

where *D* is the drug diffusivity. *k_b_* and *k_e_* refer to the elimination rate due to the blood drainage and degradation/metabolism, respectively. Two assumptions are further introduced for simplification: The linear correlation is established between the free and bound drug concentration (Eikenberry, 2009) ($K_{\mathrm{CI}}=C_{B,\mathrm{CI}}/C_{F,\mathrm{CI}};$ $K_{\mathrm{IS}}=C_{B,\mathrm{IS}}/C_{F,\mathrm{IS}}$) and the equilibrium of free drug concentration is achieved among IS, CM and CI (Saltzman & Radomsky, 1991) ($P_{\mathrm{CI}-\mathrm{IS}}=C_{F,\mathrm{CI}}/C_{F,\mathrm{IS}};$ $P_{\mathrm{CM}-\mathrm{IS}}=C_{F,\mathrm{CM}}/C_{F,\mathrm{IS}}$). As such, Equation (4) can be rewritten as (Arifin et al., 2009)

(5) $$\frac{\partial C_{F,\mathrm{IS}}}{\partial t}=D_{\mathrm{IS}}^{*}\nabla^{2}C_{F,\mathrm{IS}}-v^{*}\cdot \nabla C_{F,\mathrm{IS}}-k_{\mathrm{elim}}^{*}C_{F,\mathrm{IS}}$$

in which $D_{\mathrm{IS}}^{*}=\left(\upsilon_{\mathrm{IS}}/\omega \right)D_{\mathrm{IS}}$ is the drug apparent diffusivity, and $v^{*}=\left(\upsilon_{\mathrm{IS}}/\omega \right)v$ is the apparent interstitial fluid velocity. $k_{\mathrm{elim}}^{*}=\left[\upsilon_{\mathrm{IS}}k_{b}+\left(\upsilon_{\mathrm{IS}}+\upsilon_{\mathrm{CI}}\right)k_{e}+F_{v}\right]/\omega$ is the drug apparent elimination rate. ${F_{v}=K}_{b}\frac{S}{V}\left[p_{b}-p_{i}-\sigma_{T}\left(\pi_{b}-\pi_{i}\right)\right]$ stands for the fluid flux from blood and $\omega =\upsilon_{\mathrm{IS}}\left(1+K_{\mathrm{IS}}\right)+\upsilon_{\mathrm{CI}}P_{\mathrm{CI}-\mathrm{IS}}\left(1+K_{\mathrm{CI}}\right)+\left(1-\upsilon_{\mathrm{IS}}-\upsilon_{\mathrm{CI}}\right)P_{\mathrm{CM}-\mathrm{IS}}.$

### 2.2. Model geometry

**Figure 1.**
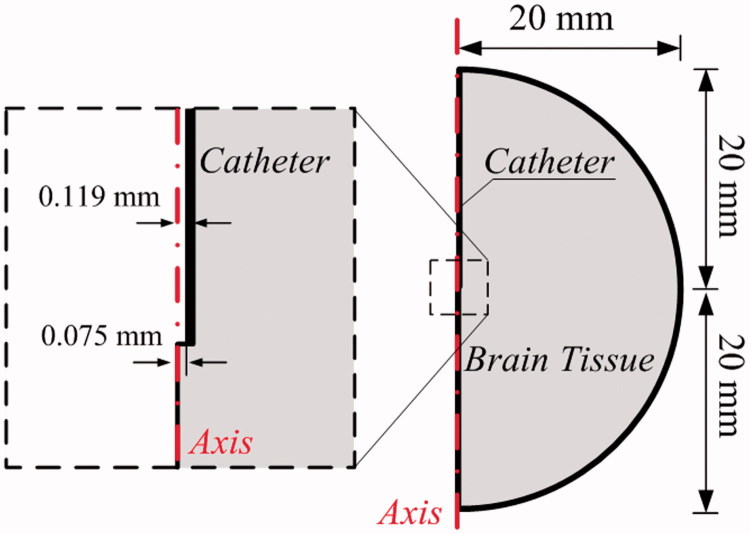
Model geometry for simulation.

The mathematical modeling of CED is performed in a 2D axis-systematic configuration (Su et al., 2011) as depicted in Figure 1. The radius of tissue domain is 20 mm (Su et al., 2010), and drugs are infused through a catheter with the inner and outer diameter of 0.15 and 0.238 mm, respectively. The final computational mesh generated in COMSOL (Su et al., 2011) consists of 50,000 triangular elements after conducting the mesh independent test. The smallest elements in 5E-3 mm are imposed on the needle wall and infusion site for the high resolution of numerical solutions.

### 2.3. Model parameters

Given that the simulation time window of CED treatments is much shorter as compared to the tissue growth, drug transport properties and geometric parameters are assumed to be independent of time. However, the variation of many parameters must still be captured in the simulations; these include physical and transport properties for the tissue and different drugs. Essential model parameters and their related sources are summarized in Table S1 and Table S2. The infusion concentration (*C*_in_) of each drug is set as its solubility, and the effective therapeutic concentration (*C*_eff_) refers to the drug dose which is sufficient to kill 90% of cultured cells as measured in *ex vivo* experiments. Justifications for the choices of the key parameters are given below.

**Infusion rate (*R_in_*)**: The infusate is administrated at a constant flow rate in CED treatments. However, the infusion rate needs to be controlled well in order to avoid potential damages to the brain tissue (Allard et al., 2009). Given this rate is recommended to be less than 5.0 μL/min (Raghavan et al., 2006; Mardor et al., 2001), it is set as 1.0 μL/min in this study.

**Tissue permeability (*κ*)**: Tissue permeability characterizes the capacity of brain tissue to enable substances’ transport through the tissue extracellular matrix. It depends on the local tissue microstructure, arrangement of nerve fibers, *etc*. Values in the range from E-15 to E-14 m^2^ were recorded and applied for the permeability of human brain tissue in different contributions (Kaczmarek et al., 1997; Linninger et al., 2008; Wagner and Ehlers, 2010; Smith et al., 2012; Vidotto et al., 2019). Hence, a mid-range value of tissue permeability, assumed to be 6.4E-15 m^2^ (Arifin et al., 2009), is used in this study.

**Drug diffusivity (*D*)**: Diffusivity represents the drug transport capacity driven by its concentration gradient in brain tissue. It is determined by several factors including the drug molecular weight, viscosity of interstitial fluid, temperature, tissue microstructure, nerve fiber arrangement, *etc*. Here, the diffusivity of *doxorubicin* is estimated based on its molecular weight and the empirical formula that was derived from the experimental measurements in Swabb et al. (1974). Values for the rest of the drugs are extracted from experiment measurements, whose details are reported in the references provided in Table S2.

**Figure 2.**
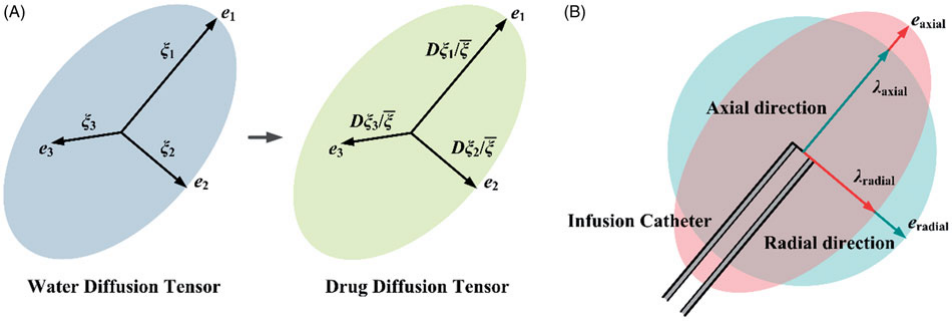
Calibration of anisotropy. (A) Derivation of anisotropy of transport properties from the local WDT. (B) Calibration procedure for parametric study.

**Calibration of anisotropy**: The anisotropy of brain tissue is usually measured by the apparent water diffusion tensor (WDT) using dMRI. As shown in Figure 2(A), given that the WDT at each pixel can be decomposed into the principal directions (***e*** *_i_*) and corresponding principal values (*ξ_i_*), the anisotropy of local transport properties (*D* and *κ*) are calculated by scaling the isotropic values with the normalized WDT principal values in each direction (Sarntinoranont et al., 2003). Following this procedure, the anisotropy of *D* and *κ* in the present study is calibrated by

(6) $$\left\{\begin{array}{c} \kappa_{\mathrm{axial}}=\kappa \lambda_{\mathrm{axial}}\\ \kappa_{\mathrm{radial}}=\kappa \lambda_{\mathrm{radial}} \end{array}\right.;\;\;\left\{\begin{array}{c} D_{\mathrm{axial}}=D\lambda_{\mathrm{axial}}\\ D_{\mathrm{radial}}=D\lambda_{\mathrm{radial}} \end{array}\right.$$

in which λ denotes how anisotropic the tissue is in the each principle direction, as shown in Figure 2(B). The angle $\theta =\mathrm{acot}\left(\lambda_{\mathrm{axial}}/\lambda_{\mathrm{radial}}\right)$ is introduced to measure the degree of local anisotropy, with $\theta =45^{o}$ corresponding to isotropic tissue.

In reality, the anisotropy of brain tissue could be considerably different depending on the local arrangements of nerve fibers (Melhem et al., 2002). Image analyses have shown that the tissue is relatively isotropic in the region close to putamen (Linninger et al., 2008); however, the maximum principle value could be 10 times higher than the minimum one near the corpus callosum (Linninger et al., 2008). Measurements based on dMRI (Linninger et al., 2008; Kim et al., 2012; Ehlers & Wagner, 2015; Dai et al., 2016) suggest that the anisotropic angle θ could vary in a wide range of (2°, 80°) throughout the entire brain.

To cover this measured range, the anisotropic angle *θ* is swept from 0° to 90° and a continuous parametric scan is performed to study the impact of diffusivity anisotropy. Given the limitation of nonzero component of *κ* as defined in Equation (2), the range of 1°∼89° is applied for tissue permeability. It is important to point out that the parameter λ is further controlled by the constrain $\sqrt{\lambda_{\mathrm{axial}}^{2}+\lambda_{\mathrm{radial}}^{2}}=1;$ this is done to feature the same baseline for cross-comparisons. However, relaxing this constrain could rescale the baseline of *D* and *κ*, and thereby divert the study focus to the magnitude of *D* and *κ* rather than their anisotropy. As the impacts of the magnitude of *D* and *κ* have been studied elsewhere, see *e.g*. Liu et al. (2010), they will not be included in this study.

### 2.4. Boundary conditions

The tissue external surface is assumed to be fixed with no displacement upon CED infusion, and the gauge pressure and drug flux are assumed to be zero (GarcÃa et al., 2013). Constant velocity and drug concentration are specified on the catheter tip, where the tissue displacement is determined by the local pressure. The tissue contacting to the catheter wall could deform depending on the local stress, and the catheter is assumed to be rigid with no slip or drug flux through its surface (Su et al., 2011).

### 2.5. Quantification index

Drug delivery outcomes in CED treatments under different conditions are evaluated in terms of drug accumulation and pharmacological effect represented by the quantitative indexes defined below.

#### 2.5.1. Effective penetration depth (*DP*_eff_)

Drug transport from the infusion site is not uniform in the brain. *DP*_eff_ is used to examine the drug effective accumulation in different directions, defined as

(7) $${\mathrm{DP}}_{\mathrm{eff}}=\sqrt{{\left(x_{i}-x_{0}\right)}^{2}+{\left(y_{i}-y_{0}\right)}^{2}}$$

where ($x_{0},\;y_{0}$) is the coordinates of the center of infusion site, and ($x_{i},\;y_{i}$) is the point where the drug concentration reduces to the effective therapeutic concentration (*C*_eff_).

#### 2.5.2. Effective delivery volume (*V*_eff_)

Treatment efficacy is evaluated in terms of the effective delivery volume, in which the drug interstitial concentration (*C_F,IS_*) in above the threshold of *C*_eff_.

(8) $$V_{\mathrm{eff}}=\sum V_{i}\;\left(C_{F,\mathrm{IS}}\geq C_{\mathrm{eff}}\right)\;$$

in which $V_{i}$ is the local element volume.

#### 2.5.3. Enhancement

A dimensionless number is introduced to access the effect of anisotropic properties on delivery outcomes in terms of either *DP*_eff_ or *V*_eff_, defined as

$\mathrm{Enhancement}=\;\frac{\text{Delivery outcomes with anisotropic properties}}{\text{Delivery outcomes with isotropic properties}}$(9)

## 3. Results

### 3.1. Drug delivery in isotropic tissue

**Figure 3.**
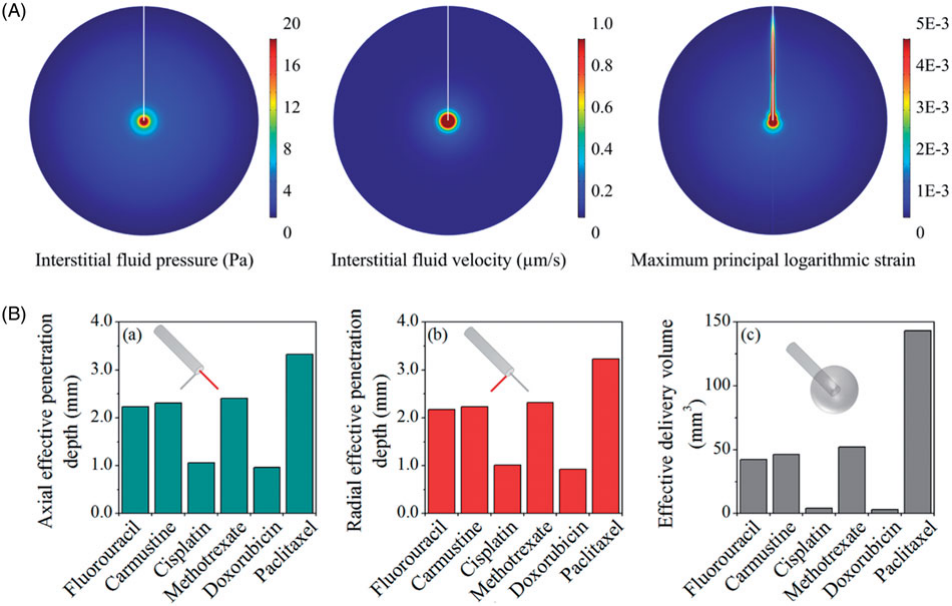
Delivery in isotropic tissue. (A) Biomechanics in brain with isotropic properties. (B) Delivery outcomes of different drugs. Penetration depth in (a) axial and (b) radial direction, and (c) distribution volume.

Drug transport and accumulation in CED treatments are strongly dependent on the mechanical environment of brain, which is predicted by solving the governing equations in the whole domain and subjected to the aforementioned boundary conditions and parameters in Table S1. Results in Figure 3(A) demonstrate that CED infusion can build up the interstitial fluid pressure around the infusion site. This raised pressure, on the one hand, is able to increase the interstitial fluid velocity and thereby enhance the drug convective transport for better drug penetration. On the other hand, the pressure difference across blood vessel wall is reduced, resulting in the reduction of fluid loss from the blood to prevent the drug concentration being diluted. The CED infusion leads to high strain around the infusion site and along the catheter track, while the impact in other regions is insignificant.

The predicted effective penetration depths (*DP*_eff_) in the axial and radial direction are compared for different drugs in Figure 3(B). Results show that *paclitaxel* presents the best penetration. It is followed by *methotrexate*, *carmustine*, *fluorouracil* and *cisplatin*, whereas the most limited penetration is found for *doxorubicin*. As a consequence, the effective delivery volumes of different drugs follow the same order.

### 3.2. Effect of anisotropic tissue permeability

The effects of anisotropic tissue permeability on the transport of each drug are examined by changing the axial and radial components with the anisotropic angle *θ* in the range from 1° to 89°, with respect to the baseline values of isotropic tissue as shown in Table S1. Other biological properties of brain tissue and transport properties of drugs are constant and isotropic.

**Figure 4.**
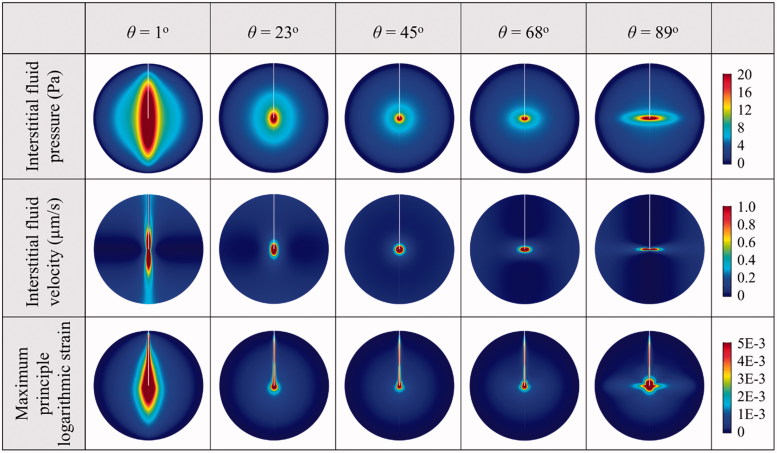
Biomechanics in brain with anisotropic tissue permeability.

The mechanical environments in the tissue with different anisotropic permeability conditions are represented in Figure 4. Comparisons show that the highest interstitial fluid pressure and tissue strain are achieved when the tissue permeability in the axial direction is dominant (*θ* = 1**°**). As a result, the interstitial fluid mainly flows along the catheter track. The interstitial fluid flow can be directed to the radial direction (as defined in Figure 2) with the increase of anisotropic angle, suggesting the flow velocity is positively related to the tissue permeability in the corresponding directions.

**Figure 5.**
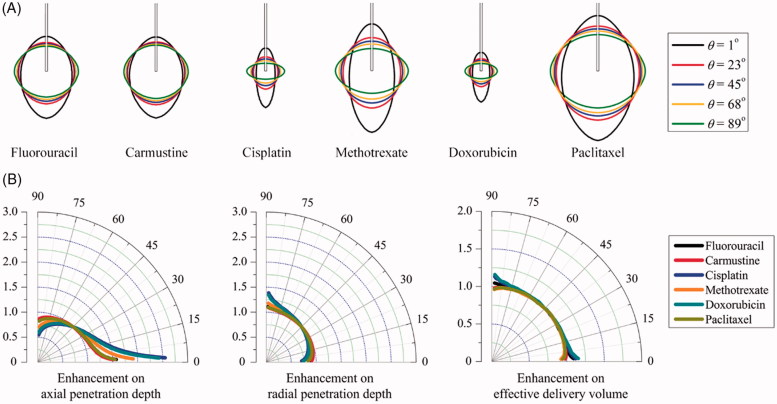
Effect of anisotropic tissue permeability on delivery outcomes of different drugs. (A) Spatial distribution of effective drug concentration. (B) Enhancement on drug effective penetration and delivery volume as a function of anisotropic angle of *θ* in the range of 1^o^ to 89^o^.

The spatial distributions of each drug in the tissues with different anisotropic tissue permeability are compared in Figure 5(A). Results demonstrate that increasing the anisotropic angle can effectively enhance the drug radial penetration, while the drug penetration in the axial direction is simultaneously reduced. The enhancements on drug penetration and delivery volume induced by the anisotropic tissue permeability are calculated based on Equation (9), and further compared in Figure 5(B) as a function of the anisotropic angle *θ*. Results denote that *cisplatin* and *doxorubicin* are the most sensitive drugs to the tissue permeability anisotropy. They are followed by *methotrexate*, *fluorouracil, paclitaxel* and *carmustine* in orders. The drug axial penetration has a more significant response to the anisotropy of tissue permeability. However, the anisotropic tissue permeability has limited impacts on the effective delivery volumes of all the examined drugs.

### 3.3. Effect of anisotropic diffusivity

**Figure 6.**
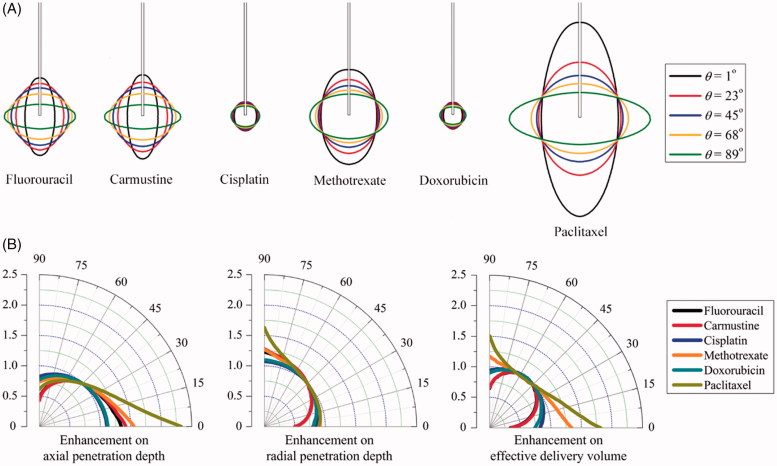
Effect of anisotropic diffusivity on delivery outcomes of different drugs. (A) Spatial distribution of effective drug concentration. (B) Enhancement on drug effective penetration and delivery volume as a function of anisotropic angle of *θ* in the range of 0^o^ to 90^o^.

The impact of anisotropic diffusivity is examined by changing the anisotropic angle in the range from 0° to 90° in this parametric study, while the rest properties are kept constant and isotropic. Modeling results of the spatial distributions of drug effective accumulation are shown in Figure 6(A). It is not surprising that the relatively uniform distribution of all drugs is achieved when the diffusivity is isotropic. Increasing the anisotropic angle can effectively increase the radial penetration, while the penetration depth in the axial direction is reduced, indicating the positive relationship between the penetration depth and the corresponding diffusivity component in the same direction.

Figure 6(B) represents the anisotropic diffusion-induced enhancements on *DP*_eff_ and *V*_eff_ as a function of *θ*. Results denote that the effective penetration of each drug present different sensitivities to the diffusivity anisotropy. As compared to *cisplatin* and *doxorubicin*, *paclitaxel* and *methotrexate* are able to transport farther into the deep region of anisotropic brain tissue. Moreover, the anisotropic diffusivity is more influential on the drug penetration in the axial direction. The enhancements on effective delivery volume are highly dependent on the drug. Modeling predictions show that the largest effective delivery volumes of *fluorouracil*, *carmustine*, *cisplatin* and *doxorubicin* can be achieved in the tissue where diffusivity is relatively isotropic, while changes in the diffusivity anisotropy could result in significant reduction in tissue volume for effective therapy. On the contrary, the delivery volume of *paclitaxel* and *methotrexate* can be largely improved by diffusivity anisotropy, with the largest coverage achieved when the drugs are diffused best in the axial direction.

## 4. Discussion

CED is designed to improve the drug delivery by means of generating a friendly mechanical environment for drug transport. On the one hand, the enhanced bulk movement of interstitial fluid flow by CED infusion can improve the convective transport of drugs in the tissue interstitial space. On the other hand, the infusion-induced high interstitial fluid pressure is capable of reducing the flow loss from blood to prevent the drug concentrations being diluted. Delivery outcomes are determined by the interplay between the drugs and the biological system. Modeling results denote that the effective penetration depth of all the examined drugs can be improved in a given direction by increasing the component of diffusivity and tissue permeability anisotropy in the same direction, which in turn requires catheters in some locations to be placed with respect to the brain matter as both diffusivity and permeability are affected by *e.g.* the distribution of axons within white matter.

Directionality of tissue response and local variations of diffusivity and permeability can now be considered when planning a neurosurgical operation. As a promising alternative to the rigid catheters that are directly inserted into the brain, a multisegment catheter has been developed (Oldfield et al., 2013) to improve the drug distribution with respect to the local anisotropic properties. The steering direction can be controlled during insertion by adjusting the relative movement of each segment. As such, this flexible catheter (Watts et al., 2019) can follow the predesigned curved trajectory (Pinzi et al., 2019) to reach the targeted location with specified orientation for drug releasing (Oldfield et al., 2014), in order to enlarge the delivery volume for improved therapy.

Here, we show how the response to anisotropic characteristics differs for different drugs. The penetration of *paclitaxel* presents high sensitivity to the diffusivity, while the changes of tissue permeability have more significant impacts on *cisplatin* and *doxorubicin*. Modeling predictions demonstrate that the tissue permeability could influence the drug penetration depth but has little contribution to the effective delivery volume. This is different from diffusivity that the treatment efficacy is strongly dependent on. Owing to the different sensitivities as shown in Figure 6, *fluorouracil*, *carmustine*, *cisplatin* and *doxorubicin* are preferable to be infused into the region where the diffusivity is less anisotropic, while *paclitaxel* and *methotrexate* are suitable to be delivered into tissue regions with significantly anisotropic diffusivity to improve the treatment efficacy.

The anisotropy of tissue permeability and drug diffusivity are usually assumed to vary in the same pattern (Linninger et al., 2008). In order to understand how the delivery outcomes of six different drugs behave in response to these anisotropic characteristics, the effects of tissue permeability and diffusivity are examined individually in this parameter study. Tissue stiffness could also be highly anisotropic in brain owing to the presence of nerve fibers. However, rather than directly determining the drug transport, anisotropic stiffness mainly influences the drug delivery by means of changing the local tissue permeability (Su et al., 2011; Kim et al., 2012). Therefore, the effect of tissue stiffness on the local tissue deformation has not been studied separately.

Simulations in this study are based on a two-dimensional axis-systematic geometry, as shown in Figure 1. Although this idealized geometry with the same dimension has been applied to study drug delivery before (Su et al., 2010), there still is a concern that the difference between the 2D idealized and 3D realistic models could significantly affect the results. To this end, additional simulations under the same delivery conditions are carried out based on a 3D brain model reconstructed from MR images, as shown in Figure S1. Results are summarized in Figure S2 and show that the modeling predictions are comparable, with very small differences obtained for all drugs adopted in this study. Therefore, the use of 2D idealized geometry is certainly acceptable for qualitatively comparing the drug delivery outcomes in the parametric study with affordable computational cost (Baxter & Jain, 1989; Su et al., 2011), while 3D models reconstructed from medical images (Zhan & Wang, 2018) may be required to obtained accurate predictions in patient-specific and more realistic clinical configurations, especially when the infusion is affected by the presence of strong gradients in local properties and tissue boundaries near the infusion site.

The feasibility of mathematical model in simulating drug delivery has been reported in several studies. The interstitial fluid velocity was calculated as 0.17 μm/s (Baxter & Jain, 1989), which was well within the experimental range of 0.13 ∼ 0.2 μm/s (Butler et al., 1975). The model-predicted interstitial fluid pressure was 40 and 1500 Pa in tumor and normal tissue, respectively (Zhan et al., 2014); the experimental measurements for these two types of tissues were −400 ∼ 800 Pa and 587 ∼ 4200 Pa (Raghunathan et al., 2010). The drug transport model was validated in gel-based experiments on CED of albumin and Evan blue. In terms of the delivery volume, the coefficients of multiple determination of 0.8 and 0.7 were achieved for each marker, respectively (Neeves et al., 2006). A similar comparison can be found in Figure S3, where the predicted concentration of bromophenol blue dye in gel agrees to the experiment. The established model is further validated by comparing with our*in vivo* experiments in Figure S4–S6. In the experiments, the gadolinium solution is continuously infused into the ovine brains at three different locations, and MR imaging is used to measure the spatial distributions. Comparisons demonstrate that the established model is capable of providing qualitative predictions of delivery outcomes. Similar validations were reported in literatures (Arifin et al., 2009; Arifin et al., 2009; Ranganath et al., 2010), where the simulated profiles of *carmustine, paclitaxel, fluorouracil and methotrexate* were found to be qualitatively comparable to the results obtained in animal experiments. By employing the transport properties calculated based on the *in vivo* data, the model-predicted accumulation profiles of nanoparticles could be quantitatively comparable to the animal experimental results (Zhan et al., 2017). In summary, modeling predictions enable comparing delivery results in a qualitative manner. The predictions can be applied to examine the impact of different factors and thereby provide opportunities to optimize the CED treatment regimes.

Although this study offers new insight into how anisotropic characteristics influence the CED treatment, the model involves several assumptions. (i) Anisotropy in the whole brain can vary significantly across the tissue; however, the local anisotropy varies gradually with respect to the continuous nerve fibers, as indicated by brain tractography (Catani et al., 2003; Lawes et al., 2008). Henceforth, the same anisotropy characteristics are assumed in the whole simulation domain that represents a small tissue region near the infusion site. This assumption can be relaxed with the additional supports of diffusion tensor imaging, from which pixel-wise anisotropic information can be acquired (Sarntinoranont et al., 2006). (ii) As shown in Figure 2(B), the infusion catheter is usually aligned with the principle directions of anisotropy. Breaking this alignment would introduce another variable in the definition of the infusion angle, which stands for the infusion orientation and catheter pose, as indicated by Figure S7. This is outside the scope of the present contribution and will be examined in future studies. (iii) The infusate concentration is set as the drug maximum solubility in water, as summarized in Table S2. Given the infusate is drug-diluted solution, the viscosity of infusate is assumed to be constant in all the simulations. However, it is worth to point out that the viscosity could be significantly changed if the infusate is suspension or emulsion. As a result, the effect of viscosity on drug accumulation and penetration should be considered. (iv) The biological properties of tissue and transport properties of drugs can also change with temperature. It is reasonable to assume the body temperature remaining at a constant level for human. However, the temperature-induced variation needs to be included for those who have inflammation or are under thermal treatments, such as ultrasound hyperthermia, *etc*. (v) Stationary simulations are performed in this study. This is because, on the one hand, the continuous CED infusion could last for several days in clinical treatments (Mardor et al., 2001); on the other hand, as a result of the equilibrium achieved between the source term of infusion and the sink term of elimination, the drug accumulation and penetration could reach a quasi-steady state after a few hours (Zhan & Wang, 2018). It is worth pointing out that the model is developed in its more general transient form, which enables predicting the spatiotemporal profiles of drug transport in CED. (vi) The drug binding with proteins and the drug partitioning in different tissue compartments are assumed to have reached equilibrium. This is because the time scale for these two-way interactions (association/disassociation and influx/efflux) is in minutes (Liu et al., 2011), while the drug penetration and accumulation could remain at the quasi-steady state for days (Mardor et al., 2001). The dynamic processes of drug binding and cell uptake can be examined by employing models reported in *e.g*. McGinty and Pontrelli (2015) and Liu et al. (2013), which the reader is invited to refer to for further details.

## 5. Conclusions

The effects of tissue permeability and drug diffusion anisotropy on the CED of six drugs have been studied based on a multiphysics model. Results demonstrate that CED infusion can successfully increase the interstitial fluid pressure and velocity around the infusion site. This hydraulic environment could be beneficial to improve the convective drug transport and reduce the fluid loss from blood circulatory system to prevent the concentration dilution. Drug penetration depth in each direction is positively related to the corresponding component of diffusivity and tissue permeability in the same direction. The anisotropic tissue permeability could effectively influence the drug spatial distribution but has limited impact on the effective delivery volume. On the contrary, the parameter study on anisotropic diffusivity shows that treatment efficacy of *fluorouracil*, *carmustine*, *cisplatin* and *doxorubicin* can be improved by infusing drugs into the relatively isotropic tissue, while the effective delivery volume of *paclitaxel* and *methotrexate* can be significantly enlarged in anisotropic tissue. Results obtained from this study can be applied as a guide for optimizing CED treatments.

## Supplementary Material

Supplemental Material
