# Supplementary material for: Effect of tissue permeability and drug diffusion anisotropy on convection-enhanced delivery
Source: Drug Deliv. 2019 Jul 30;26(1):773–81. doi: 10.1080/10717544.2019.1639844 (PMC6711026; doi:10.1080/10717544.2019.1639844)
Supplement: Supplemental Material [file IDRD_A_1639844_SM7518.docx]

***Supplementary Materials***

**Effect of Tissue Permeability and Drug Diffusion Anisotropy on Convection Enhanced Delivery**

Wenbo Zhan^*^, Daniele Dini, Ferdinando Rodriguez y Baena^*^

Department of Mechanical Engineering, Imperial College London, South Kensington Campus, London, UK

Correspondence: Dr. Wenbo Zhan, Department of Mechanical Engineering, Imperial College London, South Kensington Campus, London SW7 2AZ, UK. Tel: +44 20 7589 5111, E-mail: [w.zhan@imperial.ac.uk](mailto:w.zhan@imperial.ac.uk)

Correspondence: Professor Ferdinando Rodriguez y Baena, Department of Mechanical Engineering, Imperial College London, South Kensington Campus, London SW7 2AZ, UK. Tel: +44 207594 7046, E-mail: [f.rodriguez@imperial.ac.uk](mailto:f.rodriguez@imperial.ac.uk)

**S1. Comparison of simulations using 2D idealised and 3D realistic geometric model**

Although the 2D idealised axis-systematic geometry has been applied in simulating drug delivery in previous studies, see *e.g.* Ref. [[1](#_ENREF_1)], there still is a concern that the difference between a 2D idealised model and a 3D model obtained from image reconstruction could affect the results of drug delivery simulations. Therefore, additional simulations are carried out based on a 3D realistic brain extracted from patient MR images, as shown in **Figure S1**.

**
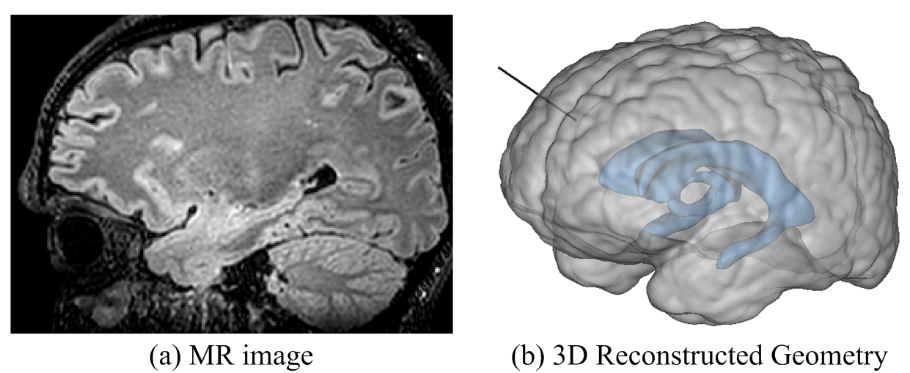
**

**Figure S1. 3D realistic model geometry. (a) A representative MR image, and (b) the 3D reconstructed geometry of brain. The ventricle, brain tissue and infusion catheter are marked in grey, blue and black, respectively. All procedures performed in this study involving human participants were in accordance with the ethical standards of the ethics committee of the San Raffaele-Milan hospital (80/INT/2016).**

The 2D and 3D simulations are run under the same infusion parameters and tissue isotropic properties as summarised in **Table S1** and **Table S2** [[2](#_ENREF_2)]. Representative pressures of 1447.4 Pa [[3](#_ENREF_3)] and 657.9 [[4](#_ENREF_4)] Pa are imposed on the surfaces of ventricle and brain tissue [[5](#_ENREF_5)], respectively. The infusion site is also chosen to be representative of brain tumour clinical procedures.

The modelling results using the 2D and 3D models are shown in **Figure S2**. Cross-comparisons on the penetration depth and delivery volumes demonstrate that the modelling predictions of using these two models are comparable. The very small differences, which are slightly more pronounced for *Paclitaxel* as this drug has the largest infusion volume, can be attributed to the boundary conditions imposed to the model. Whilst in the 2D model drugs are administrated in the centre of the domain (note that two different 2D models are used to show that the size of the domain does not affect the results beyond a 20 mm radius), distances from the infusion site to brain surface are not uniform in the 3D model and large infusion volumes will start to be affected by the free boundary corresponding to the brain surface. As a consequence, the impact of the boundary conditions to drug delivery differs with respect to the infusion location in the two models. However, cross-comparisons demonstrate that this impact is relatively small, and finding indicates that the 2D idealised model is applicable in the parametric study.





**Figure S2. Comparisons of modelling predictions using 2D idealised model and 3D realistic brain models on effective penetration in (a) axial direction, (b) radial direction and (c) effective distribution volume. Two 2D idealised models are used, with the radius of 20mm and 40mm, respectively.**

**S2. Comparison of modelling predictions with gel-based experiments**

Results available from the literature were used to verify the validity of the infusion model. An *in silico* experiment was performed by simulating the delivery of bromophenol blue dye into a gel to replicate experimental data obtained in Ref. [[6](#_ENREF_6)]. The 3.7E-3 M dye solution was infused through the 27G needle at the infusion rate of 1.0 μL/min for 30 min. The validation study is carried out using the same infusion protocol. Results in **Figure S3** shows that the predicted drug concentration profile agrees with the experimental data, demonstrating the validity of the mathematical model for simulating drug delivery in CED treatments.


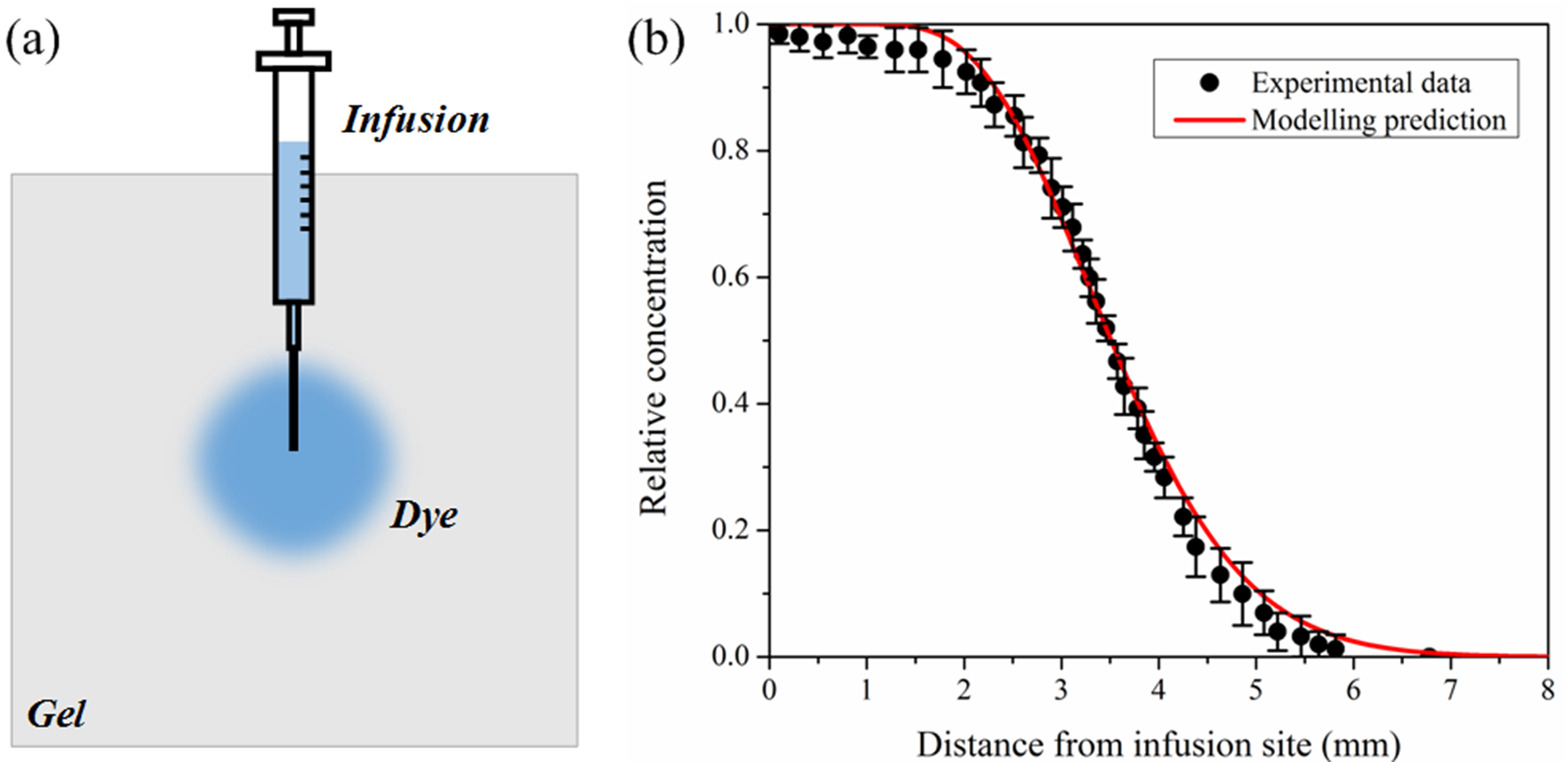


**Figure S3. Model validation: (a) Schematic of the *in silico* experiments. (b) Comparison of concentration profiles as a function of the distance from infusion site. The effective diffusivity of bromophenol blue dye is 1.6E-10 m^2^/s** [[6](#_ENREF_6)]**, and experimental data are extracted from Ref.** [[6](#_ENREF_6)].

**S3. Comparison of modelling predictions to animal experiments**

The modelling predictions are compared with ovine experiments in **Figure S4**, **S5** and **S6** in which the infusion location differs. In each experiment, a total volume of 10 μL gadolinium solution is infused at a constant rate 3.0 μL/min into the ovine brain. The infusate concentration is 6.5 mol/m^3^. The gadolinium diffusivity in isotropic tissue is calculated as 1.55E-10 m^2^/s [[7](#_ENREF_7)], and its elimination rate is 0.01 min^-1^ [[8](#_ENREF_8)]. The tissue anisotropy is measured by diffusion tensor imaging (DTI) at each pixel, and registered to the 3D reconstructed ovine brain models for simulations.

The spatial distributions of gadolinium in the experiments are measured by MR imaging as shown **Figure S4(a)**, **S5(a)** and **S6(a)**. The corresponding modelling predictions at the same time points are given in **Figure S4(b)**, **S5(b)** and **S6(b)**, respectively. The gadolinium distribution is found to be relatively isotropic in Case_1 from both the experimental and modelling results, as represented in **Figure S4 (a)** and **(b)**. To be different, gadolinium is more penetrable along the infusion direction in Case_2, shown in **Figure S5 (a)** and **(b)**. The similar pattern can also be found in **Figure S6 (a)** and **(b)**, where gadolinium mainly transports in the direction normal to the infusion in Case_3.

In summary, the modelling predictions could qualitatively agree with the experimental measurements using MR imaging. This finding indicates that the established mathematical model is capable of demonstrating the impact of tissue anisotropy on the drug delivery using CED.


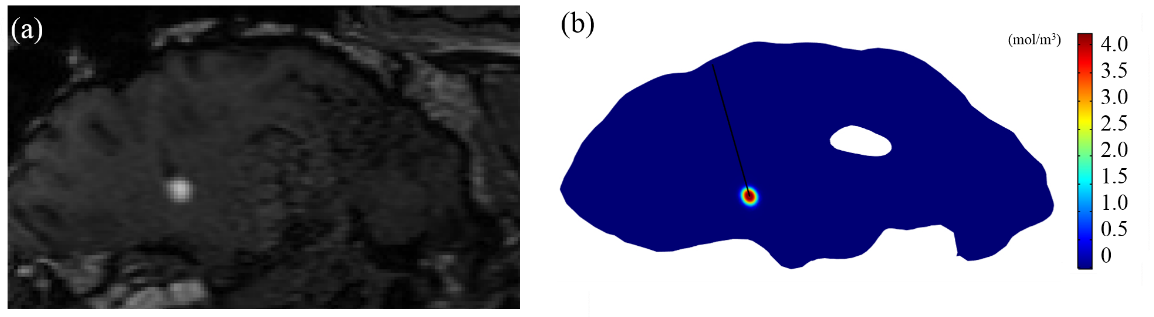


**Figure S4. Model validation with animal experiment Case_1: (a) MR image (*t*=20min), (b) modelling prediction (*t*=20min). All procedures performed in studies involving ovine experiments were in accordance with the ethical standards of the ethics committee of the San Raffaele-Milan hospital (80/INT/2016).**

**
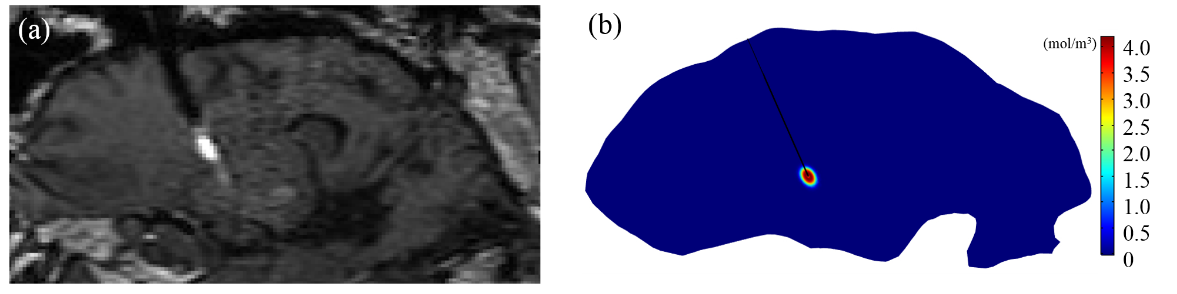
**

**Figure S5. Model validation with animal experiment Case_2: (a) MR image (*t*=10min), (b) modelling prediction (*t*=10min). All procedures performed in studies involving ovine experiments were in accordance with the ethical standards of the ethics committee of the San Raffaele-Milan hospital (80/INT/2016).**

**
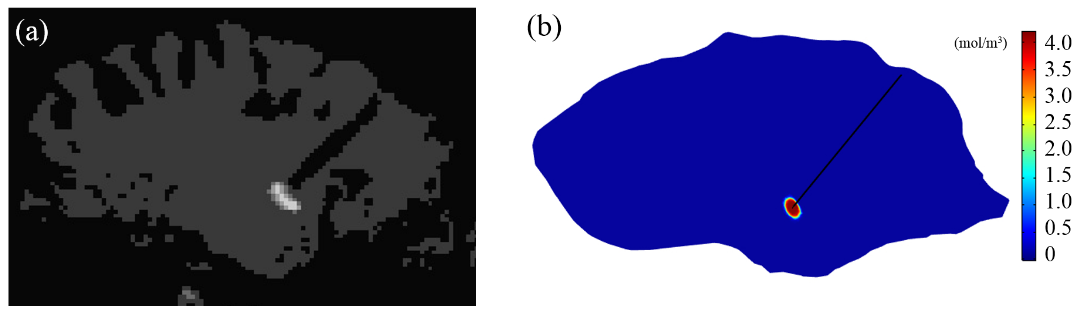
**

**Figure S6. Model validation with animal experiment Case_3: (a) MR image (*t*=20min), (b) modelling prediction (*t*=20min). All procedures performed in studies involving ovine experiments were in accordance with the ethical standards of the ethics committee of the San Raffaele-Milan hospital (80/INT/2016).**

**S4. Placement of infusion catheter**

In this parametric study, the catheter is placed by aligning the infusion direction with the principal direction of local anisotropy. Breaking this alignment would require the inclusion of another variable, infusion angle (*Φ*), which stands for the catheter orientation and pose, as demonstrated in **Figure S7**. As this study is focused on the impact of tissue anisotropy, the variation of the catheter placement is not considered here and will be addressed in future studies.

**
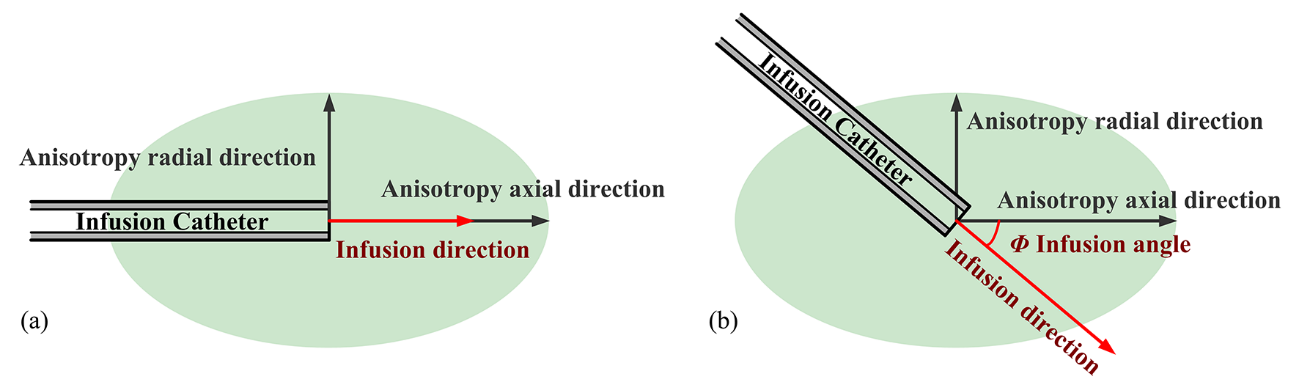
**

**Figure S7. The placement of infusion catheter with respect to the principal direction of local anisotropy. (a) The infusion direction aligns with the principal direction of anisotropy, (b) there is an angle between the infusion direction and anisotropy principal direction.**

**Table S1. Parameters for brain tissue.**

| Parameter | Unit | Value |
| --- | --- | --- |
| *υ_IS_* | - | 0.35 [[9](#_ENREF_9)] |
| *υ_CI_* | - | 0.55 [[9](#_ENREF_9)] |
| *ρ* | kg/m^3^ | 1000 [[10](#_ENREF_10)] |
| *μ* | Pas | 7.8E-4 [[10](#_ENREF_10)] |
| *π_b_* | Pa | 3440 [[3](#_ENREF_3)] |
| *π_i_* | Pa | 1110 [[11](#_ENREF_11)] |
| *p_b_* | Pa | 4610 [[3](#_ENREF_3)] |
| *λ* | Pa | 4286 [[12](#_ENREF_12)] |
| *G* | Pa | 1071 [[12](#_ENREF_12)] |
| *S/V* | m^-1^ | 20000 [[11](#_ENREF_11)] |
| *σ_T_* | - | 0.82 [[11](#_ENREF_11)] |
| *L_b_* | m/Pa/s | 1.1E-12 [[13](#_ENREF_13)] |
| *M* | - | 2.0 [[12](#_ENREF_12)] |
| *κ_0_* | m^2^ | 5.0E-15 [[13](#_ENREF_13)] |

**Table S2. Transport properties of chemotherapeutic drugs.**

| Parameter | Unit | Fluorouracil | Carmustine | Cisplatin | Methotrexate | Doxorubicin | Paclitaxel |
| --- | --- | --- | --- | --- | --- | --- | --- |
| *P_CI-IS_* | - | 1.0 [[14](#_ENREF_14)] | 1.0 [[14](#_ENREF_14)] | 1.0 [[14](#_ENREF_14)] | 1.0 [[14](#_ENREF_14)] | 1.0 [[14](#_ENREF_14)] | 1.0 [[14](#_ENREF_14)] |
| *P_CM-IS_* | - | 0.1 [[15](#_ENREF_15)] | 10.3 [[14](#_ENREF_14)] | 0.006 [[16](#_ENREF_16)] | 0.01 [[15](#_ENREF_15)] | 0.3 [[17](#_ENREF_17)] | 3162.3 [[18](#_ENREF_18)] |
| *K_IS_, K_CI_* | - | 0.1 [[19](#_ENREF_19)] | 5.0 [[14](#_ENREF_14)] | 1.0 [[20](#_ENREF_20)] | 0.7 [[21](#_ENREF_21)] | 3.0 [[22](#_ENREF_22)] | 5.1 [[23](#_ENREF_23)] |
| *D_IS_* | m^2^/s | 1.2E-9 [[15](#_ENREF_15)] | 1.5E-9 [[14](#_ENREF_14)] | 2.5E-10 [[24](#_ENREF_24)] | 5.3E-10 [[15](#_ENREF_15)] | 3.4E-10 [[25](#_ENREF_25)] | 9.0E-10 [[18](#_ENREF_18)] |
| *k_b_* | s^-1^ | 1.8E-2 [[15](#_ENREF_15)] | 1.4E-2 [[14](#_ENREF_14)] | 2.9E-2 [[24](#_ENREF_24)] | 2.8E-4 [[15](#_ENREF_15)] | 6.0E-2 [[25](#_ENREF_25)] | 1.4E-4 [[18](#_ENREF_18)] |
| *k_e_* | s^-1^ | 5.6E-4 [[15](#_ENREF_15)] | 1.1E-4 [[14](#_ENREF_14)] | 7.3E-4 [[24](#_ENREF_24)] | 1.5E-4 [[15](#_ENREF_15)] | 5.8E-4 [[26](#_ENREF_26)] | 6.8E-7 [[18](#_ENREF_18)] |
| *C_in_* | M | 7.7E-3 [[27](#_ENREF_27)] | 1.9E-2 [[28](#_ENREF_28)] | 3.3E-3 [[29](#_ENREF_29)] | 3.7E-4 [[30](#_ENREF_30)] | 1.8E-3 [[30](#_ENREF_30)] | 7.0E-6 [[31](#_ENREF_31)] |
| *C_eff_* | M | 2.0E-6 [[32](#_ENREF_32)] | 1.5E-5 [[18](#_ENREF_18)] | 2.0E-5 [[32](#_ENREF_32)] | 5.9E-5 [[32](#_ENREF_32)] | 2.4E-6 [[33](#_ENREF_33)] | 8.9E-7 [[18](#_ENREF_18)] |

**References**

[1] J.J. GarcÃa, A.B. Molano, J.H. Smith, Description and validation of a finite element model of backflow during infusion into a brain tissue phantom, Journal of Computational and Nonlinear Dynamics, 8 (2013) 011017.

[2] W. Zhan, M. Alamer, X.Y. Xu, Computational modelling of drug delivery to solid tumour: Understanding the interplay between chemotherapeutics and biological system for optimised delivery system, Advanced Drug Delivery Reviews, (2018) 81-103.

[3] H. Kimelberg, Water homeostasis in the brain: basic concepts, Neuroscience, 129 (2004) 851-860.

[4] J.F. Gross, A.S. Popel, Mathematical models of transport phenomena in normal and neoplastic tissue, CRC Press, Boca Raton, FL, USA1979.

[5] W. Zhan, Delivery of liposome encapsulated temozolomide to brain tumour: Understanding the drug transport for optimisation, International journal of pharmaceutics, 557 (2019) 280-292.

[6] A.A. Linninger, M.R. Somayaji, L. Zhang, M.S. Hariharan, R.D. Penn, Rigorous mathematical modeling techniques for optimal delivery of macromolecules to the brain, IEEE Transactions on Biomedical Engineering, 55 (2008) 2303-2313.

[7] E.A. Swabb, J. Wei, P.M. Gullino, Diffusion and convection in normal and neoplastic tissues, Cancer research, 34 (1974) 2814-2822.

[8] T. Nhan, A. Burgess, L. Lilge, K. Hynynen, Modeling localized delivery of Doxorubicin to the brain following focused ultrasound enhanced blood-brain barrier permeability, Physics in Medicine & Biology, 59 (2014) 5987-6004.

[9] S. Kalyanasundaram, V. Calhoun, K. Leong, A finite element model for predicting the distribution of drugs delivered intracranially to the brain, American Journal of Physiology-Regulatory, Integrative and Comparative Physiology, 273 (1997) R1810-R1821.

[10] D.W. Green, R.H. Perry, Perry's Chemical Engineers' Handbook/edición Don W. Green y Robert H. Perry, 1973.

[11] L.T. Baxter, R.K. Jain, Transport of fluid and macromolecules in tumors. I. Role of interstitial pressure and convection, Microvascular research, 37 (1989) 77-104.

[12] D. Su, R. Ma, L. Zhu, Numerical study of nanofluid infusion in deformable tissues for hyperthermia cancer treatments, Medical & biological engineering & computing, 49 (2011) 1233-1240.

[13] D.Y. Arifin, K.Y.T. Lee, C.-H. Wang, Chemotherapeutic drug transport to brain tumor, Journal of controlled release, 137 (2009) 203-210.

[14] L.K. Fung, M. Shin, B. Tyler, H. Brem, W.M. Saltzman, Chemotherapeutic drugs released from polymers: distribution of 1, 3-bis (2-chloroethyl)-l-nitrosourea in the rat brain, Pharmaceutical research, 13 (1996) 671-682.

[15] W.M. Saltzman, M.L. Radomsky, Drugs released from polymers: diffusion and elimination in brain tissue, Chemical Engineering Science, 46 (1991) 2429-2444.

[16] T.-L. Hwang, C.-L. Fang, C.-H. Chen, J.-Y. Fang, Permeation enhancer-containing water-in-oil nanoemulsions as carriers for intravesical cisplatin delivery, Pharmaceutical research, 26 (2009) 2314-2323.

[17] T. Formariz, V. Sarmento, A. Silva-Junior, M. Scarpa, C.V. Santilli, A. Oliveira, Doxorubicin biocompatible O/W microemulsion stabilized by mixed surfactant containing soya phosphatidylcholine, Colloids and Surfaces B: Biointerfaces, 51 (2006) 54-61.

[18] L.K. Fung, M.G. Ewend, A. Sills, E.P. Sipos, R. Thompson, M. Watts, O.M. Colvin, H. Brem, W.M. Saltzman, Pharmacokinetics of interstitial delivery of carmustine, 4-hydroperoxycyclophosphamide, and paclitaxel from a biodegradable polymer implant in the monkey brain, Cancer Research, 58 (1998) 672-684.

[19] D.S. Wishart, C. Knox, A.C. Guo, S. Shrivastava, M. Hassanali, P. Stothard, Z. Chang, J. Woolsey, DrugBank: a comprehensive resource for in silico drug discovery and exploration, Nucleic acids research, 34 (2006) D668-D672.

[20] W. Cole, W. Wolf, Preparation and metabolism of a cisplatin/serum protein complex, Chemico-biological interactions, 30 (1980) 223-235.

[21] M. Maia, S. Saivin, E. Chatelut, M. Malmary, G. Houin, In vitro and in vivo protein binding of methotrexate assessed by microdialysis, International journal of clinical pharmacology and therapeutics, 34 (1996) 335-341.

[22] R.F. Greene, J.M. Collins, J.F. Jenkins, J.L. Speyer, C.E. Myers, Plasma pharmacokinetics of adriamycin and adriamycinol: implications for the design of in vitro experiments and treatment protocols, Cancer research, 43 (1983) 3417-3421.

[23] H.-J. Kuh, S.H. Jang, M.G. Wientjes, J.L.-S. Au, Computational model of intracellular pharmacokinetics of paclitaxel, Journal of Pharmacology and Experimental Therapeutics, 293 (2000) 761-770.

[24] D.K. Shah, B.S. Shin, J. Veith, K. Tóth, R.J. Bernacki, J.P. Balthasar, Use of an anti-vascular endothelial growth factor antibody in a pharmacokinetic strategy to increase the efficacy of intraperitoneal chemotherapy, Journal of Pharmacology and Experimental Therapeutics, 329 (2009) 580-591.

[25] W. Zhan, W. Gedroyc, X.Y. Xu, Effect of heterogeneous microvasculature distribution on drug delivery to solid tumour, Journal of Physics D: Applied Physics, 47 (2014) 475401.

[26] Y.-M.F. Goh, H.L. Kong, C.-H. Wang, Simulation of the delivery of doxorubicin to hepatoma, Pharmaceutical Research, 18 (2001) 761-770.

[27] A. Buur, H. Bundgaard, Prodrugs of 5-fluorouracil. III. Hydrolysis kinetics in aqueous solution and biological media, lipophilicity and solubility of various 1-carbamoyl derivatives of 5-fluorouracil, International journal of pharmaceutics, 23 (1985) 209-222.

[28] P.B. Layton, H.S. Greenberg, P.L. Stetson, W.D. Ensminger, J.W. Gyves, BCNU solubility and toxicity in the treatment of malignant astrocytomas, Journal of neurosurgery, 60 (1984) 1134-1137.

[29] W.-P. Liu, Q.-S. Ye, Y. Yu, X.-Z. Chen, S.-Q. Hou, L.-G. Lou, Y.-P. Yang, Y.-M. Wang, Q. Su, Novel lipophilic platinum (II) compounds of salicylate derivatives, Platinum Metals Review, 52 (2008) 163-171.

[30] I.V. Tetko, J. Gasteiger, R. Todeschini, A. Mauri, D. Livingstone, P. Ertl, V.A. Palyulin, E.V. Radchenko, N.S. Zefirov, A.S. Makarenko, Virtual computational chemistry laboratory–design and description, Journal of computer-aided molecular design, 19 (2005) 453-463.

[31] R.T. Liggins, W. Hunter, H.M. Burt, Solid‐state characterization of paclitaxel, Journal of pharmaceutical sciences, 86 (1997) 1458-1463.

[32] C.M. Hand, J.R. Vender, P. Black, Chemotherapy in experimental brain tumor, part 1: in vitro colorimetric MTT assay, Journal of neuro-oncology, 36 (1998) 1-6.

[33] D.J. Kerr, A.M. Kerr, R.I. Freshney, S.B. Kaye, Comparative intracellular uptake of adriamycin and 4'-deoxydoxorubicin by nonsmall cell lung tumor cells in culture and its relationship to cell survival, Biochemical pharmacology, 35 (1986) 2817-2823.
